# Galanin ameliorates liver inflammation and fibrosis in mice by activating AMPK/ACC signaling and modifying macrophage inflammatory phenotype

**DOI:** 10.3389/fimmu.2023.1161676

**Published:** 2023-04-26

**Authors:** Lingnan He, Chao Huang, Hui Wang, Naibin Yang, Jianbin Zhang, Leiming Xu, Ting Gu, Zhenghong Li, Yuanwen Chen

**Affiliations:** ^1^ Department of Gastroenterology, Huadong Hospital Affiliated to Fudan University, Shanghai, China; ^2^ Department of Geriatrics, Huadong Hospital Affiliated to Fudan University, Shanghai, China; ^3^ Endoscopy Center, Department of Gastroenterology, Shanghai East Hospital, Tongji University School of Medicine, Shanghai, China; ^4^ Department of Gastroenterology, Xinhua Hospital, Shanghai Jiaotong University School of Medicine, Shanghai, China; ^5^ Department of Gastroenterology, Shanghai Tenth People’s Hospital, Tongji University School of Medicine, Shanghai, China; ^6^ Department of Endoscopic, Affiliated Tumor Hospital of Zhengzhou University, Henan Cancer Hospital, Zhengzhou, Henan, China; ^7^ Department of Infectious Diseases, Ningbo First Hospital, Ningbo Hospital of Zhejiang University, Ningbo, Zhejiang, China

**Keywords:** galanin, non-alcoholic steatohepatitis, liver fibrosis, macrophage, AMPK

## Abstract

**Background and aims:**

Galanin is a naturally occurring peptide that plays a critical role in regulating inflammation and energy metabolism, with expression in the liver. The exact involvement of galanin in non-alcoholic fatty liver disease and related fibrosis remains controversial.

**Methods:**

The effects of subcutaneously administered galanin were studied in mice with non-alcoholic steatohepatitis (NASH) induced by a high-fat and high-cholesterol diet for 8 weeks, and in mice with liver fibrosis induced by CCl_4_ for 7 weeks. The underlying mechanism was also studied *in vitro* on murine macrophage cells (J774A.1 and RAW264.7).

**Results:**

Galanin reduced inflammation, CD68-positive cell count, MCP-1 level, and mRNA levels of inflammation-related genes in the liver of NASH mice. It also mitigated liver injury and fibrosis caused by CCl_4_. *In vitro*, galanin had anti-inflammatory effects on murine macrophages, including reduced phagocytosis and intracellular reactive oxygen species (ROS). Galanin also activated AMP-activated protein kinase (AMPK)/acetyl-CoA carboxylase (ACC) signaling.

**Conclusion:**

Galanin ameliorates liver inflammation and fibrosis in mice, potentially by modifying macrophage inflammatory phenotype and activating AMPK/ACC signaling.

## Graphical Abstract

Galanin ameliorates NASH in mice, potentially by modifying macrophage inflammatory phenotype. AMPK/ACC signaling mediates the effect of galanin on shifting inflammatory phenotype of macrophages.

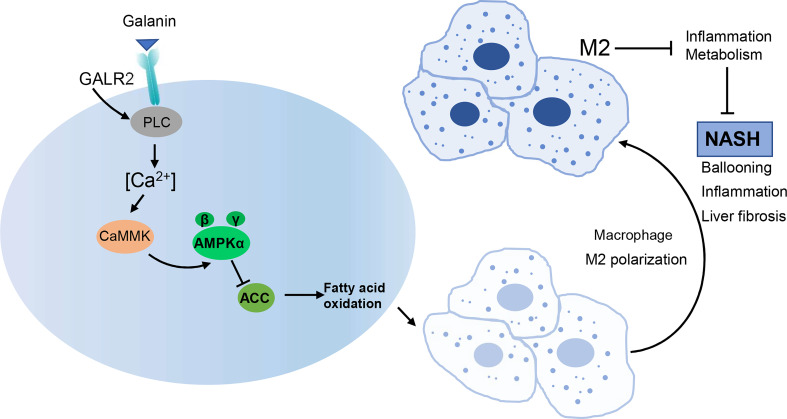

## Introduction

Galanin, a 29-amino-acid neuropeptide, has been shown to have a widespread distribution in central nervous system and peripheral tissues (1). The galanin receptors have three subtypes: GalR1, GalR2 and GalR3 (2), which belong to G protein-coupled receptors, and signal *via* multiple transduction pathways, including inhibition of cyclic AMP/protein kinase A (GalR1, GalR3) and stimulation of phospholipase C (GalR2) (3). This explains why one specific molecule of galanin can be responsible for different roles in different tissues. Galanin is induced in inflammation and exerts anti-inflammatory effects *via* certain galanin-receptor subtypes (4). The results of galanin on inflammation vary based on the disease and the dominant receptors on affected organ cells. This highlights the complexity of galanin’s role in inflammation regulation. In addition, galanin is also associated with metabolism. It affects food intake and energy expenditure (5), as well as inhibits insulin and leptin secretion (6) (7). Galanin integrates the signals of fat stores, nutrient intake and energy expenditure to regulate energy homeostasis (8). In response to sympathetic stimulation, large amounts of galanin are produced from the liver and released into the systemic circulation (9). However, its biological effect on liver remains unclear.

Non-alcoholic fatty liver disease (NAFLD) refers to a clinicopathologic syndrome characterized by fat accumulation in the liver parenchymal cells due to insulin resistance and factors excluding alcohol (10). The disease spectrum of NAFLD includes simple fatty liver (SFL), steatohepatitis (NASH) and related liver fibrosis (11). NASH is the critical phase in NAFLD progression, while inflammatory responses are well-known as the key step from SFL to NASH (12). Therefore, investigating the inflammation in this phase in the liver and insulin resistance is particularly important.

The relationship between galanin and NAFLD pathogenesis *in vivo* remains inconclusive (8). Galanin plays a role in glucose regulation, inhibiting insulin release while promoting insulin sensitivity in skeletal muscle, heart muscle, and adipose tissue (13, 14). The role of galanin in liver fibrosis and inflammation is also complex, with some studies suggesting that it can induce scar formation in the liver, while others indicate that targeting the galanin pathway may have therapeutic potential in treating fatty liver disease (13–15). Interestingly, our previous research observed that galanin could inhibit hepatic stellate cell (HSCs) activation and suppress the profibrogenic feature of HSCs by activating GalR2 (16).

Therefore, in this study, we explored the changes in galanin levels in patients during the development of NAFLD and examined the effects of galanin in the initial stage of NASH in a mouse model induced by a high-fat and high-cholesterol diet (HFHCD). We also explored the effects of galanin on liver fibrosis by infusion of exogenous galanin into the CCl_4_-treated mice. Mechanistically, galanin activates AMPK signaling and promotes M2-polarization in macrophages. Our data uncover insights into function of galanin in NAFLD and provide a molecular basis for therapeutic strategies targeting altered metabolic phenotype in NAFLD.

## Materials and methods

### Patient specimens

Blood samples were obtained from 62 patients with NAFLD at Xinhua Hospital following the approved IRB protocol. Sixty-two patients with NAFLD were confirmed with liver ultrasound. Three grades of steatosis have been proposed (grade 1: liver echogenicity increased; grade 2: the echogenic liver obscuring the echogenic walls of the portal venous branches; grade 3: the echogenic liver obscuring the diaphragmatic outline). Meanwhile, the blood of 38 normal adults was also collected. The age, heart rate, body mass index(kg/m^2^), waist circumference (cm), systolic blood pressure (mmHg), and diastolic blood pressure (mmHg) of each patient were recorded. The alanine aminotransferase (ALT), total cholesterol, triglycerides(mmol/L), fasting blood glucose, galanin, leptin, and insulin were detected from each blood sample.

### Animal models

Male C57BL/6 mice (aged 7–8 weeks) were obtained from Slack Company (Shanghai, China). All animal handling and experimental procedures were in accordance with the ethical standards of Xinhua Hospital Ethics Committee Affiliated to Shanghai Jiaotong University School of Medicine (Shanghai, China).

#### High-fat and high-cholesterol diet induced NASH models

Forty mice were randomly divided into three groups. Mice received a HFHCD (composition: 88% standard chow, 10% lard, 2% cholesterol) or a standard chow diet (Control). The effects of galanin (Bachem Co., Switzerland) were evaluated by injecting it subcutaneously (abdominal area, 8 µg/100 g body weight) daily from the beginning of the ninth week of dietary intervention for 5 weeks (17). Blood was collected before all mice were sacrificed by cervical dislocation after overnight fasting. Tissue samples were either directly snap-frozen in liquid nitrogen for molecular analysis or fixed in 4% paraformaldehyde and embedded in paraffin for histological analysis.

#### CCl_4_-induced liver fibrosis models

Forty mice were randomly divided into three groups. Liver fibrosis was induced by subcutaneous injection of a 2:3 solution of CCl_4_ and olive oil (2.5ul/g body weight) twice per week (CCl_4_ model) for 7 weeks as previously described. The effects of galanin were evaluated by injecting it subcutaneously (abdominal area, 10 µg/100 g body weight) daily. Upon sacrifice, blood was collected and serum and/or plasma were obtained. Liver tissue was either fixed in paraformaldehyde liquid, frozen in optimal cutting temperature, or snap-frozen in liquid nitrogen and stored at -80°C.

### Macrophage cell lines cultures

Murine macrophage cell lines J774A.1 (ATCC TIB67) and RAW264.7 were cultured in Dulbecco’s modified eagle medium (DMEM) (100U/ml of penicillin and 100µg/ml of streptomycin) containing 10% fetal bovine serum (FBS) at 37°C in a humidified 5% CO_2_ atmosphere.

### Histological analysis and Immunohistochemistry

Paraformaldehyde-fixed paraffin sections were stained with hematoxylin-eosin (HE) for pathological analysis. Masson’s trichrome staining was performed to assess liver fibrosis, and sections were stained with Masson’s trichrome stain kit (Solarbio, shanghai, China) according to the manufacturer’s instructions.

After dewaxing and hydration, the sections were incubated overnight with antibodies against CD68 (ab125212, Abcam, Hong Kong). Sections were incubated with biotinylated secondary antibodies for 30 minutes. The reaction was visualized by 3,3’-Diaminobenzidine (DAB). Slides were counterstained with hematoxylin, dehydrated with sequential ethanol, hyalinized with dimethylbenzene and sealed with neutral gum. CD68-positive cells were manually counted in 10 randomly selected, non-overlapping fields.

The NAFLD activity score (NAS) was assessed based on steatosis, intralobular inflammation, and ballooning hepatocyte degeneration (18). Six sections were randomly selected from each group and observed under an electron microscope (Olympus, Japan, 400× magnification).

### Serum analysis

The blood was centrifuged at 3000r/min, for 20 minutes. Serum ALT was measured using an automated analyzer. ELISAs were used to detect galanin (Bachem Co.), insulin (Alpco Immunoassays, USA), leptin and monocyte chemotactic protein-1 (MCP-1) (R&D SYSTEMS, USA) in strict accordance with the operating instructions for each product.

### Quantitative real-time PCR

Total RNA was extracted using Trizol and was reverse-transcribed using a verse Transcription system (Code No.: RR036A, TAKARA, Japan). The RNA concentration was measured using a NanoDrop spectrophotometer, and 1μg of RNA was used for cDNA synthesis for each sample. Quantitative real-time PCR (qRT-PCR) of 2 ul cDNA was performed with TB Green Premix Ex Taq (Tli RNaseH Plus) (Code No.: RR420A, TAKARA, Japan). The primer sequences for amplification of each gene are listed in **Supplemental Table S1**. GAPDH was the internal control.

### Western blot analysis

Western blot analysis was performed as previously described (19). Cells or tissues were lysed in cold RIPA buffer containing protease inhibitors. Equal amounts of total protein were separated by SDS–PAGE, transferred to PVDF membrane, and analyzed by immunoblotting. Membranes were probed with the following antibodies: anti-iNOS (ab178945, Abcam), anti-Arg1(ab233548, Abcam), anti-AMPKα (#9957, CST), anti-p-AMPKα(Thr172) (#9957, CST), anti-ACC (#9957, CST), anti-p-ACC (#9957, CST), anti-*a*-SMA(A5228, Sigma). Anti-rabbit-HRP and anti-mouse-HRP (Beyotime Biotechnology, China) were used as secondary antibodies.

### Cell proliferation assay

Cell proliferation assays were performed using Cell Counting Kit-8 (Dojindo, Kumamoto, Japan) following manufacturer’s instructions. The cell numbers in triplicate wells were measured as the absorbance (450 nm) of reduced blank.

### Phagocytic activity of cells

The phagocytotic activity of J774A.1 and RAW264.7 cells was assessed as described previously with minor modifications (20). Quiescent cells (serum-starvation 4h) were challenged by lipopolysaccharide (LPS) (0.1µg/ml) for 2 hours and then exposed to various concentrations of galanin for 24 hours. The latex beads (10%, 3µm size, Sigma) were added and incubated for an additional 4 hours. The percentage of cells containing three or more latex beads was determined by counting 200 cells.

### Fluorescent measurement of intracellular reactive oxygen species

J774A.1 and RAW264.7 cells were incubated overnight in DMEM containing 10% FBS. Cells were loaded with 5µM 2,7-Dichlorodihydrofluorescein diacetate (DCFH-DA) in phosphate balanced solution (PBS) for 20 minutes at 37°C. The fluorescence was monitored after 5 minutes, using excitation and emission wavelengths of 485nm and 530nm, respectively.

### Transwell for migration assay

J774A.1 and RAW264.7 cells were seeded in 6-well plates and cultured for 3 days. Cell migration assay was performed in a transwell Boyden chamber (Corning). Cells suspension (3×10^5^ cells/mL) was placed in the upper chamber with serum-free medium. The lower compartment contained 0.6 ml DMEM containing 10% FBS. After 24 hours of incubation at 37°C, the cells and DMEM in the upper chamber were removed. The chamber was fixed with 4% paraformaldehyde and then stained with 0.5% crystal violet (Beyotime) for 20 minutes. The cells in 5 different fields were photographed and counted using a microscope.

### Hydroxyproline content

Hydroxyproline content in liver tissue was determined using acid hydrolysis method (21). Liver tissues (0.5 g) were hydrolyzed (20 hours in 6 mol/L HCl at 100°C), diluted in ultrapure water, and centrifuged to eliminate contaminants. Samples were incubated for 10 minutes in 0.05 mol/L chloramine-T at room temperature, followed by a 15-minute incubation in Ehrlich’s perchloric acid solution at 65°C. The absorbance was measured at 561 nm, and the value for each sample was computed using a hydroxyproline standard curve. Date expressed in milligrams of hydroxyproline per gram of wet liver tissue.

### Statistical analysis

The results are expressed as mean ± SD. Statistical analyses were performed by using ANOVA with the statistical software GraphPad Prism 7. *Post hoc* Student–Newman–Kuels analyses were performed when >2 groups were present.

## Results

### Galanin is increased in the serum of patients with NAFLD

**Figure 1:**
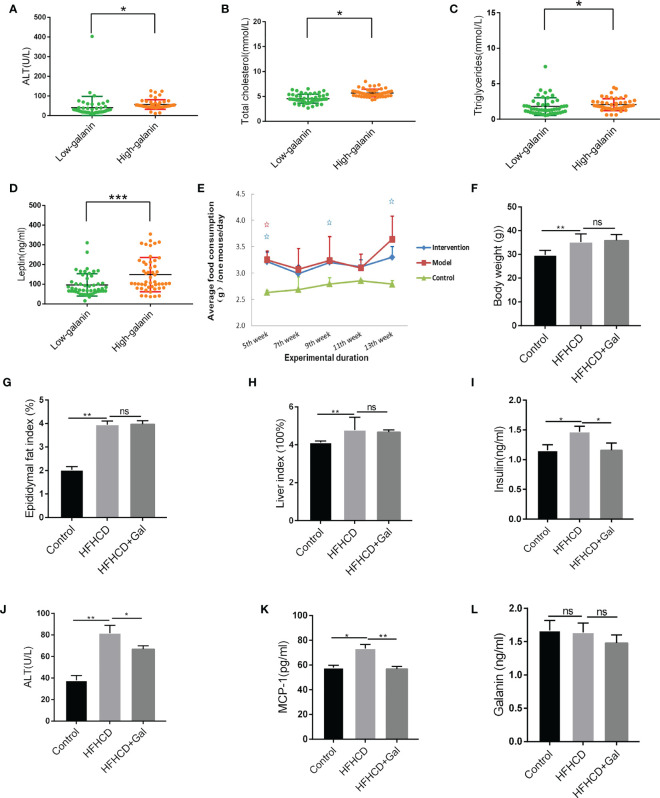
Galanin alters metabolism in HFHCD-fed mice. **(A)** Correlation analysis of ALT and galanin in human samples. *, p < 0.05. **(B)** Correlation analysis of total cholesterol and galanin in human samples. *, p < 0.05. **(C)** Correlation analysis of triglycerides and galanin in human samples. *, p < 0.05. **(D)** Correlation analysis of leptin and galanin in human samples. ***, p < 0.001. **(E)** The feed consumption of mice in each group. **(F–H)** The body weight **(F)**, epididymal fat index (epididymal fat weight g/weight g) **(G)** and liver index (liver weight g/weight g) **(H)** in HFHCD-fed mice. **, p < 0.01. **(I–L)** The serum insulin **(I)**, ALT **(J)**, MCP-1 **(K)** expression and serum galanin levels **(L)** in HFHCD-fed mice. *, p < 0.05, **, p < 0.01. ALT: Glutamic-pyruvic transaminase. MCP-1: Monocyte chemotactic protein -1.

To investigate the difference in galanin content between NAFLD and normal controls, we detected serum galanin from 62 NAFLD patients and 38 normal adults. The serum galanin was upregulated in NAFLD patients in comparison with normal control group(p=0.028). NAFLD patients showed higher body mass index (p <0.0001) and abdominal circumference (p <0.0001) (**Table 1**). More importantly, a high galanin content was associated with increased ALT content (**Figure 1A**), upregulated total cholesterol (**Figure 1B**), more elevated triglycerides

**Table 1:** Galanin levels in patients with NAFLD.

	NAFLD(n=62)	Control(n=38)	*P* value
Sex(M/F)	46/16	15/25	-
Age(years)	43.73 ± 1.30	33.89 ± 10.37	<0.0001
Heart rate	81.04 ± 1.54	82.08 ± 2.68	NS 0.736
Body mass index (kg/m^2^)	25.88 ± 0.410	20.81 ± 0.489	<0.0001
Waist circumference (cm)	82.307 ± 0.913	71.395 ± 0.937	<0.0001
Systolic blood pressure (mmHg)	131.84 ± 2.216	115.58 ± 2.340	<0.0001
Diastolic blood pressure (mmHg)	86.47 ± 1.578	77.13 ± 1.530	<0.0001
Alanine aminotransferase (U/L)	43.565 ± 6.724	19.13 ± 1.689	0.001
Total cholesterol (mmol/L)	12.476 ± 0.703	4.221 ± 0.118	<0.0001
Triglycerides(mmol/L)	1.920 ± 0.147	1.209 ± 0.094	<0.0001
Fasting blood glucose(mmol/L)	5.817 ± 0.223	5.216 ± 0.082	0.042
Galanin(ng/ml)	362.39 ± 19.558	309.11 ± 13.715	0.028
Leptin(ng/ml)	147.921 ± 10.674	81.253 ± 6.616	<0.0001
Insulin(U/ml)	23.861 ± 2.137	13.064 ± 2.137	0.001

(**Figure 1C**) and leptin (**Figure 1D**). Serum galanin was not associated with other parameters, such as gender and heart rate.

### Galanin alters metabolism in HFHCD–induced mouse NASH models

To explore the therapeutic potential of galanin in NASH, we treated HFHCD-fed mice with galanin. We first evaluated the overall metabolic status of mice. HFHCD-fed mice received additional treatment of galanin showed no significant difference in the food consumption, body weight, epididymal fat index (epididymal fat weight g/weight g) and liver index (liver weight g/weight g) compared with HFHCD-fed mice (**Figures 1E–H**).

In HFHCD-fed mice, galanin treatment repressed serum insulin, ALT and MCP-1 expression, suggesting metabolism changes and decreased tissue damage in these animals (**Figures 1I–K**). In addition, there were no significant differences in serum galanin levels among all groups (**Figure 1L**).

### Chronic exogenous galanin infusion attenuated NASH development and fibrosis

**Figure 2:**
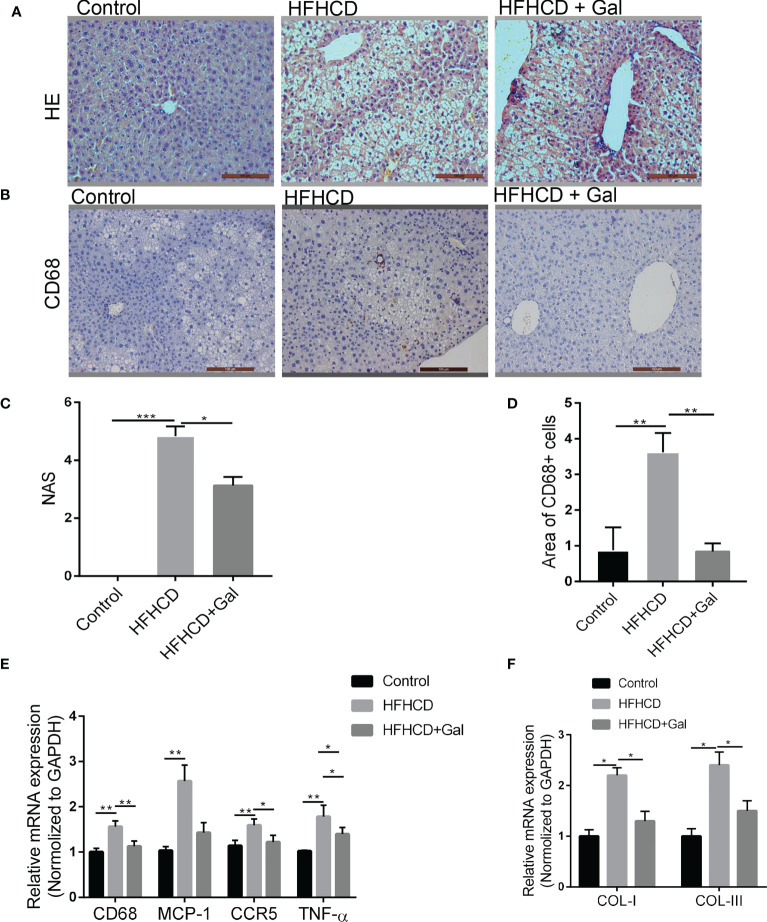
Galanin improves liver inflammation in HFHCD–fed mice. **(A)** HE staining on representative liver tissue sections from mice in each group. **(B)** Immunohistochemical analysis of CD68 on representative liver tissue sections from mice in each group. **(C)** NAS analysis using electron microscope; 6 sections per mouse were quantified. *, p < 0.05, ***, p < 0.001. **(D)** Quantitative analysis of **(B)** **, p < 0.01. **(E)** CD68, MCP-1, CCR5 and TNF-α mRNA levels of liver assessed by qRT-PCR. *, p < 0.05, **, p < 0.01. **(F)** Liver gene expressions of COL-I and COL-III in each group. *, p < 0.05. HE, Hematoxylin-eosin staining; NAS, NAFLD activity score; CCR5, C-C chemokine receptor type 5; TNF-α, Tumor necrosis factor-α; GAL, Galanin.

The livers of HFHCD-fed mice exhibited varying degrees of hepatic steatosis, intralobular infiltration of inflammatory cells, loose cytoplasm and ballooning degeneration of hepatocytes. As shown in **Figures 2A, C**, galanin treatment decreased the mouse liver NAS in HFHCD-fed mice. Kupffer cells (KCs) are hepatic macrophages that play a pivotal role in the key steps of fatty liver progression to fibrosis (22), with CD68 as a surface marker. CD68 immunostaining confirmed KCs infiltration in HFHCD-fed mice, which was reduced by galanin treatment (**Figures 2B, D**). In keeping with this observation, the mRNA levels of CD68 and those of MCP-1, a potent macrophage chemoattractant, were significantly higher in the livers of HFHCD-fed mice than in control mice(p<0.01), and this difference disappeared following treatment with galanin(p<0.01). The hepatic expression of other inflammatory markers such as chemokine C-C-motif receptor 5(CCR5) and tumor necrosis factor (TNF)-α mRNA were significantly increased in HFHCD-fed mice than in control mice (p<0.01). This difference was abrogated or reduced following treatment with galanin(p<0.01) (**Figure 2E**). Features of liver fibrosis were detected in HFHCD-induced NASH models. The results revealed that galanin reduced collagen I (COL-I) and collagen III (COL-III) mRNA in NASH models (**Figure 2F**).

**Figure 3:**
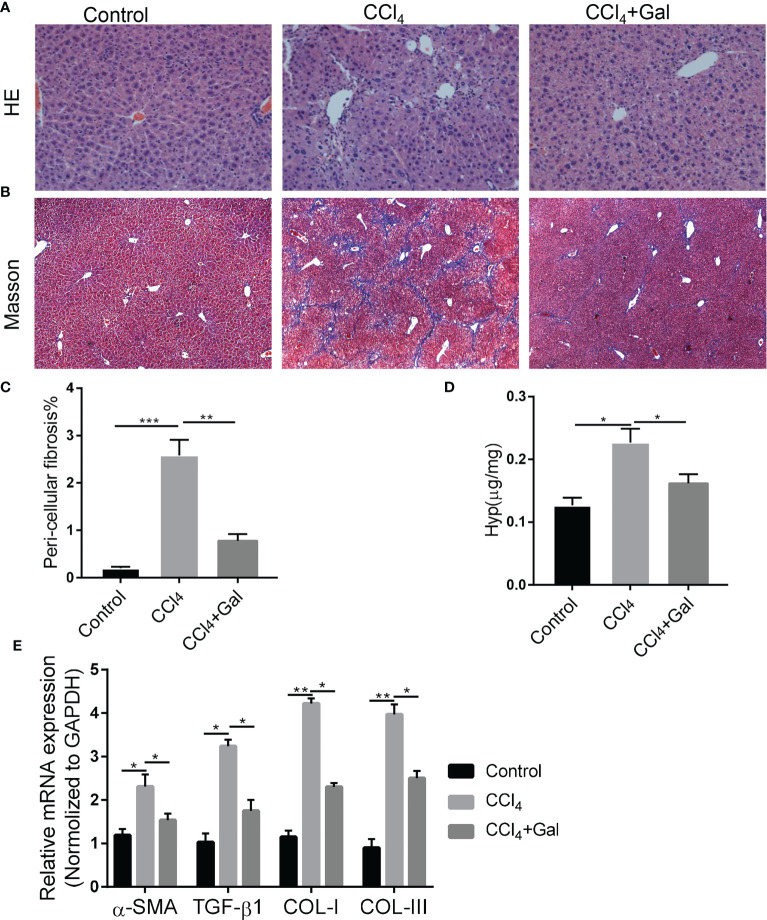
Galanin improves fibrosis induced by CCl_4_. **(A, B)** Representative images of liver sections stained with HE and Masson. **(C)** Histological quantification of liver fibrosis. **, p < 0.01. **(D)** Hydroxyproline content in liver in each group. *, p < 0.05. **(E)** Liver gene expressions of α-SMA, TGF-β1, COL-I and COL-III in each group. *, p < 0.05, **, p < 0.01. α-SMA, Alpha smooth muscle actin; TGF-β1, Transforming growth factor beta1; COL-I, Type I collagen; COL-III, Type III collagen; GAL, Galanin.

To further explore the role of galanin in fibrosis induced by chronic liver injury and inflammation, we treated mice with carbon tetrachloride (CCl_4_) or its vehicle for 7 weeks (23). HE staining of liver tissue sections showed that CCl_4_-fed mice developed pericellular fibrosis, which was abrogated following galanin treatment. Masson staining also revealed that marked collagen accumulation was observed in CCl_4_–treated mice, which were attenuated by galanin infusion (**Figures 3A–C**). Likewise, increases in hydroxyproline content observed in the livers of CCl_4_–treated mice were downregulated by galanin infusion (**Figure 3D**). Liver mRNA levels of fibrogenesis markers (α-SMA, TGF-β1, COL-I and COL-III) were also increased in CCl_4_-fed mice. They were all downregulated by galanin treatment (**Figure 3E**). These data conclusively demonstrated that treatment with galanin prevents the histologic features of NASH and fibrosis.

### Galanin inhibits pro-inflammatory phenotype of murine macrophages induced by LPS *in vitro*

**Figure 4:**
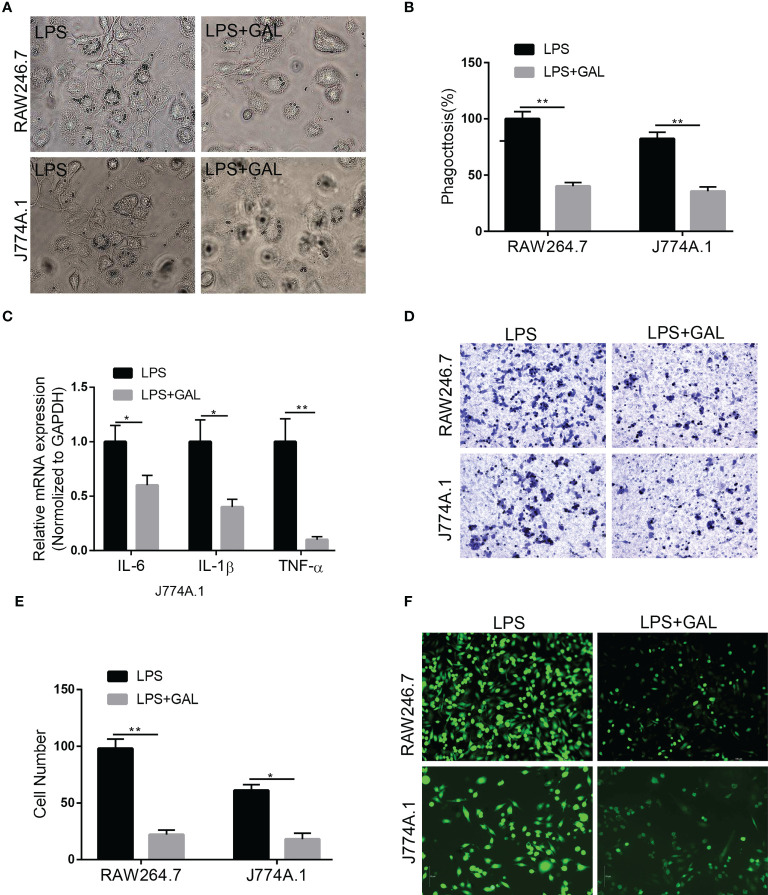
Galanin improves inflammation in murine macrophages. Murine macrophages were incubated with LPS to induce inflammation, and treated with or without galanin(1000nmol/L). **(A)** Representative images showing the internalization of latex beads by J774A.1 and RAW264.7 cells. **(B)** Quantitative analysis of **(A)** **, p < 0.01. **(C)** IL-6, IL-1β and TNF-α mRNA levels in J774A.1 macrophages assessed by qRT-PCR. *, p < 0.05, **, p < 0.01. **(D)** Transwell assay on J774A.1 and RAW264.7 macrophages to investigate the effect of galanin on migration. **(E)** Quantification of **(D)** *, p < 0.05. **, p < 0.01. **(F)** Representative image of detecting intracellular ROS by using the peroxide-sensitive probe DCFH-DA. IL-6, Interleukin-6; IL-1β, Interleukin-1β; DCFH-DA, 2’,7’-Dichlorodihydrofluorescein diacetate; GAL, Galanin.

**Figure 5:**
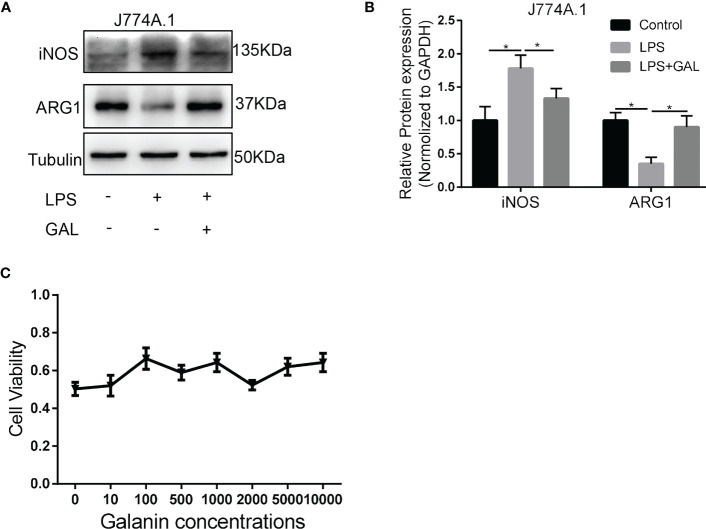
Galanin induces M2-polarization phenotype in murine macrophages. Murine macrophages were incubated with LPS to induce inflammation, or without (Control) and treated with or without galanin(1000nmol/L). **(A)** Representative image of western blot analysis of iNOS and ARG1 expression in J774A.1 macrophages. **(B)** Quantification of **(A)** *, p < 0.05. **(C)** CCK-8 assay of each concentration of galanin on J774A.1 macrophage proliferation. LPS, Lipopolysaccharide; iNOS, Inducible Nitric Oxide Synthase; ARG1, Arginase 1; GAL, Galanin.

To understand how galanin regulates inflammatory role of macrophage, we assess the effect of galanin on macrophage function upon LPS challenge (1 μg/ml for 2 hours). Cells challenged by LPS showed potent phagocytic activity, which was alleviated by galanin administration (**Figures 4A, B**). The initially elevated mRNA level of inflammatory cytokines (TNF-α, IL-6 and IL-1β) under LPS challenge showed a significant decrease following treatment with galanin (**Figure 4C**). Galanin also weakened macrophage cell migration after LPS challenge (**Figures 4D, E**). In addition, using the peroxide-sensitive probe DCFH-DA, we found that galanin induced a significant decrease in intracellular ROS production in macrophage cells (**Figure 4F**). Galanin-treated macrophages underwent a marked decrease in inducible nitric oxide synthase (iNOS) and a significant increase in Arginase 1(Arg1) compared to LPS challenge alone (**Figures 5A, B**). This effect was independent of cell proliferation (**Figure 5C**). The galanin receptors were detected in macrophages, revealing that macrophages expressed GalR2 (**Supplementary Figure 1**). These findings suggest galanin’s novel function in blocking pro-inflammatory phenotype of macrophages expressing GalR2.

### AMPK/ACC signaling mediates the effect of galanin on shifting pro-inflammatory phenotype of macrophages

**Figure 6:**
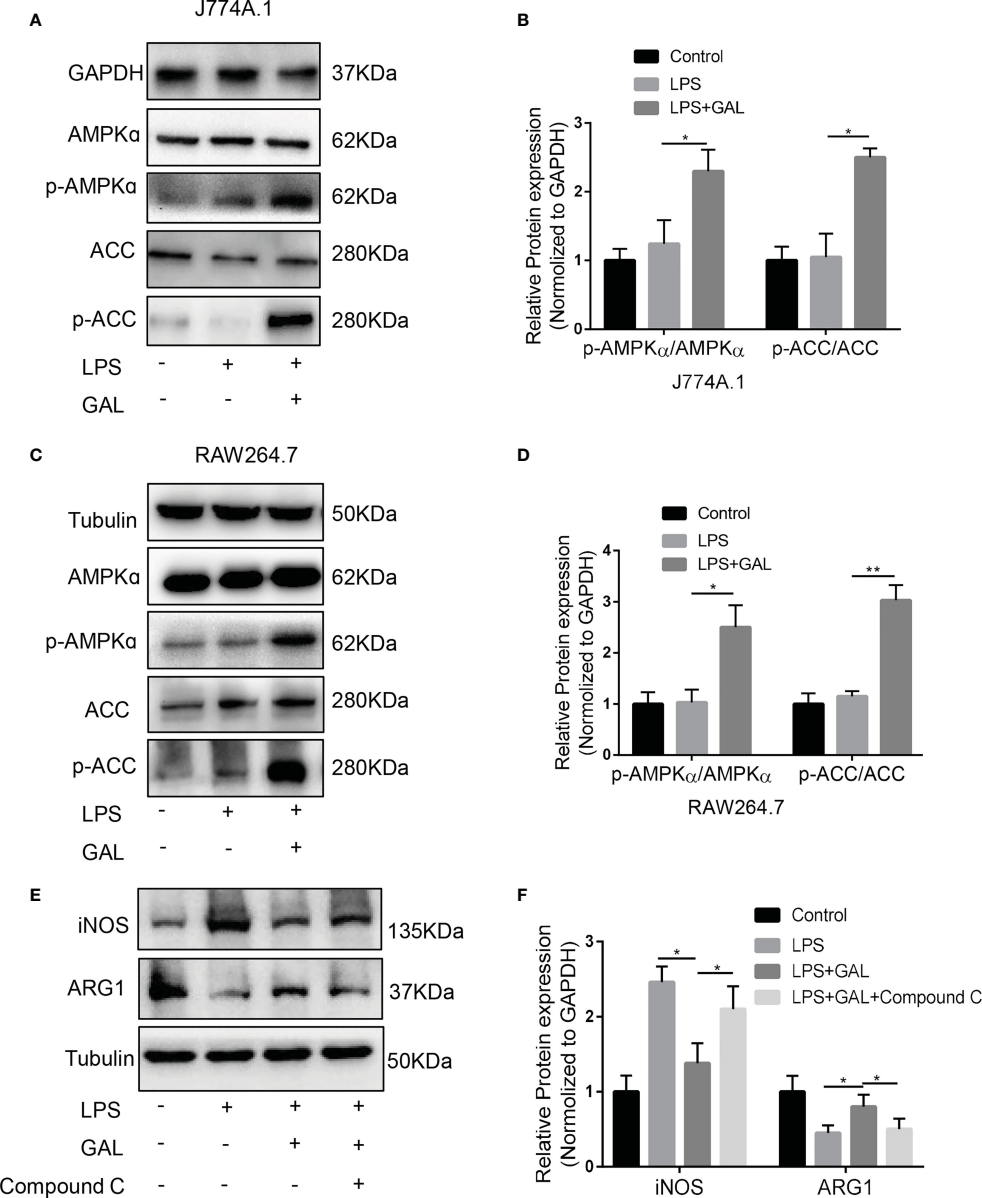
AMPK/ACC signaling mediates the effect of galanin on shifting inflammatory phenotype of macrophages. **(A)** The protein levels of AMPK, p-AMPK, ACC, p-ACC expressions in response to galanin in J774A.1 macrophages were measured by western blot. **(B)** Quantification of **(A)** *, p < 0.05. **(C)** The protein levels of AMPK, p-AMPK, ACC, p-ACC expressions in response to galanin in RAW264.7 macrophages were measured by western blot. **(D)** Quantification of **(C)** *, p < 0.05. **, p < 0.01. **(E)** J774A.1 macrophages were pretreated with Compound **(C)** iNOS and ARG1 expressions were detected by western blot. **(F)** Quantification of **(E)** *, p < 0.05. AMPK: AMP-activated protein kinase. p-AMPK, Phosphorylated AMP-activated protein kinase; ACC, Acetyl-CoA carboxylase; p-ACC, Phosphorylated acetyl-CoA carboxylase; GAL, Galanin.

From our studies, galanin not only causes metabolism changes but also opposes inflammation in NASH. Insight into the role of metabolism in inflammation is supported by recent findings concerning the role of adenosine 5’-monophosphate (AMP)-activated protein kinase (AMPK) as a key signaling pathway to contact inflammation and metabolism (24). To further explore the underlying mechanism of how galanin regulates pro-inflammatory phenotype of macrophages, we evaluate the effect of galanin on AMPK activation. We observed that under LPS challenge, galanin treatment induced AMPK phosphorylation on Thr172 and phosphorylates the downstream mediator acetyl-CoA carboxylase (ACC) in macrophages (**Figures 6A–D**). To confirm whether the anti-inflammatory effects of galanin on macrophages were dependent on the activation of AMPK signaling pathway, macrophages were pretreated with Compound C, a selective AMPK inhibitor. The results showed that the M2-polarization induced by galanin on macrophages was partially blocked after treatment of Compound C (**Figures 6E, F**). Collectively, these findings demonstrate that galanin might promote M2 polarization of macrophages through AMPK activation.

## Discussion

This study provides evidence suggesting that galanin improves histologic features of NASH including liver inflammation and fibrosis. Moreover, galanin shift inflammatory phenotype of macrophages. In addition, we show that AMPK signaling pathway mediates galanin’s anti-inflammatory effects in macrophages. These data suggest that galanin could be therapeutic in NAFLD/NASH and NASH fibrosis.

The major finding in the present study was the observation that galanin alleviates NASH in mice. NASH is a potential outcome of NAFL, a condition that occurs when lipids accumulate in hepatocytes (25). Inflammation is the key rate-limiting step for NAFL to develop into NASH (26). The importance of the galanin family of peptides as inflammatory modulators is supported by data obtained from several experimental models of inflammation. The galanin family modulates inflammation in the peripheral tissues and may regulate immunity as galanin expression can be altered in inflammatory conditions (27–29). Studies indicate that galanin switches off the inflammatory response by regulating innate immunity mechanisms such as proinflammatory cytokine production (4, 30). Here we demonstrated that galanin is increased in NAFLD patients and is correlated to proinflammatory factors in NASH, including MCP-1 and leptin, suggesting a protective responder to NASH inflammation, even though early studies also suggested a deleterious effect of galanin in obesity and hepatic steatosis. Our investigation in galanin-treated mice model of NASH supported the protective effect of galanin in NASH development.

Kupffer cells are the macrophages in the liver and play a pivotal role in initiating and perpetuating the inflammatory response, with a major deleterious impact on key steps of fatty liver progression to fibrosis (31). Our results showed that galanin significantly attenuated macrophage infiltration and hepatic inflammation in HFHCD-fed mice. This aligns with previous studies that galanin modulated the expression of chemokines and cytokine levels in macrophages (32). These findings further support the concept that the body responds to NASH inflammation by increasing the production of galanin peptides to switch off the inflammatory response and restore immune homeostasis, rather than exacerbating the inflammatory response (4).

Homozygous galanin transgenic mice demonstrated increased body weight and development of metabolic syndrome (33). In our study, galanin seemingly improves lipid metabolism in liver without regulating food intake and body weight, which is consistent with published studies that galanin was not associated with body weight, food intake and/or fat intake in humans (34). Nevertheless, these suggest that peripherally administered galanin is closer to the actual pathophysiology in the animal than transgenic mice. Non-selective galanin transgenic over-expression animals have long and high levels of circulating galanin, which might result in action on the central nervous system to promote food intake. However, due to the short circulating time of galanin in our study, it is unlikely to pass through the blood-brain barrier to affect the feeding center.

Interestingly, one study showed that chronic administration of oral galanin once daily in diabetic mice increased insulin sensitivity and improved several metabolic parameters such as glucose tolerance, fasting blood glucose, and insulin. The researchers concluded that oral galanin administration improves glucose homeostasis *via* the enteric nervous system, indicating its potential as a therapeutic for treating T2D. Strikingly, they did not observe a significant variation of plasma galanin in response to this oral treatment once daily, suggesting that galanin’s effect on glucose metabolism is short-term and related to the intestine (15). A similar phenomenon was observed in our study where an abdominal subcutaneous injection of galanin once daily did not increase circulating galanin but was protective against NAFLD progression. Whether or not a similar mechanism was involved in our study still needs further investigation.

Hepatic fibrosis is an advanced stage of NAFLD progression and HSCs are the key matrix-producing cells in liver and play a central role in hepatic fibrogenesis (35). Our previous study demonstrated that galanin inhibits HSCs activation and suppresses their profibrogenic features (16). In the present study, our research further confirmed that galanin had preventive effect on CCl_4_-induced liver fibrosis *in vivo*, and improved liver function. Therefore, galanin is a promising endogenous factor involved in inhibiting NASH and fibrosis. Nonetheless, the mechanism by which transient changes in galanin are transformed into a favorable effect is still unclear and needs further investigation in future studies.

The biological activity of galanin is mediated *via* specific receptors, and the effects of galanin may differ depending on the dominant receptor(s) in different diseases and cell types (8). Studies in mice have shown that galanin treatment can increase cholangiocyte proliferation and fibrogenesis in liver fibrosis of biliary damage induced by multidrug resistance protein 2 knockout (Mdr2KO) (9). Conversely, suppressing galanin receptors has been found to reduce bile duct mass and hepatic fibrosis. In biliary hyperplasia induced by bile duct ligation in rats, galanin has been found to contribute to cholangiocyte proliferation (36). This discrepancy with our study may be due to differences in disease conditions and the galanin receptor activated.

In peripheral tissues and cells, such as adipose tissue, macrophages, and hepatocytes, the primary galanin receptor is GalR2/3. Galanin can exert its anti-inflammatory effects *via* galanin-receptor subtypes, mainly GalR2/3, while GalR1 was thought to be pro-inflammatory and pro-fibrogenic in the liver (4, 8). In these animal models of bile duct injury, the major effects are induced *via* activation of galanin receptor 1 (GalR1), which is expressed specifically on cholangiocytes, while HSCs and hepatocytes express GalR2 (9, 36) (16).

Spexin, a neuropeptide in the galanin family, has been shown to activate GALR2/GALR3 (but not GALR1) and mitigate diet-induced hepatic steatosis *in vivo* and *in vitro* by activating GALR2. Studies have demonstrated that these beneficial effects were eliminated by the GALR2 antagonist M871 in mice fed a high-fat diet and in palmitic acid-induced HepG2 cells, highlighting the critical role of the GALR2 signaling pathway in spexin’s ability to improve fatty liver disease. Furthermore, spexin was found to activate GAL2 receptors and relieve skeletal muscle insulin resistance while improving metabolic parameters and adipocyte hypertrophy in obese mice by reducing M1 macrophages and subtypes and improving adipose tissue inflammation (37–40).

Macrophages are a crucial determinant for the progression of NAFLD (41). We have found that galanin regulates the inflammatory phenotype of macrophages, leading to a shift toward M2 polarization. Our results demonstrated that macrophages from LPS challenged exhibited an M1 polarized phenotype. However, galanin increased the expression of Arg1 and reduced the expression of the M1 macrophage marker iNOS in macrophages. It could be that galanin induced M2 polarization and reduced M1 polarization. Arg1 production is increased in M2-polarized macrophages. This enzyme blocks iNOS activity by various mechanisms, including competing for the arginine substrate required for nitroxide production (42, 43). In adipose and liver, M2 macrophages are usually immunoregulatory and maintain insulin sensitivity, whereas M1 macrophages disrupt insulin sensitivity (44). This may explain why galanin decreased the number of CD68 positive cells in NASH in the present study.

Metabolic changes in cells that participate in inflammation, such as activated macrophages, include a shift towards enhanced glucose uptake, glycolysis and increased pentose phosphate pathway activity (45). Altered metabolism may thus participate in the signal-directed programs that promote or inhibit inflammation (46). Recent studies have shown that AMPK is a key signal in modulating inflammatory responses in immune cells and regulating lipid and glucose metabolism (47, 48). Acetyl-CoA carboxylase (ACC), a downstream target of activated AMPK, is vital in regulating fatty acid oxidation in the liver (49). AMPK phosphorylates ACC1/2 on serine residues (Ser79/212), leading to inhibition of ACC activity (50) and decreased fatty acid synthesis (51), and increases the oxidation of fatty acid, reduced lipid storage in the liver (52). Consistent with this, we observed the activation of AMPK/ACC signaling in macrophages induced by galanin. Mechanically, activation of macrophages GalR2 is able to activate PLC activity (53), mediating the release of Ca2+ into the cytoplasm from intracellular stores and opening Ca2+-dependent channels. AMPK has been demonstrated as a multifunctional anti-inflammatory protein and can be activated *via* Ca2+/CaMKKβ pathway (54). Therefore, galanin may activate PLC activity by GalR2 in macrophages, mediating AMPK signaling *via* Ca2+/CaMKKβ pathway. It suggests that galanin activating AMPK-ACC signaling pathway might be associated with its anti-inflammatory effect. It has been revealed that metabolic reprogramming changes in hepatic macrophages play an essential role in macrophage phenotype shift (55). M2-polarized macrophages express Arg1 and convert l-arginine to urea and ornithine, competing with iNOS, which is highly expressed in M1-polarized macrophages and converts it into NO (56). The anti-inflammatory role of galanin — based on the activation of AMPK-ACC signaling pathway— seems to have been in response to metabolism changes in macrophages. As previously reported, oral administration of galanin increases total and phosphorylated AMPK protein expression, favoring glucose flux through glycolysis and activating AMPK signaling in the liver and muscles (15).

Our study has some limitations. Firstly, only male mice were used, and sex may be a crucial experimental variable in mice models. However, previous research has shown that male mice exhibit more severe liver fibrosis and inflammation with a high-fat diet (57). Future studies will include male and female mice to explore potential sex-specific differences in the effects of galanin on NASH and fibrosis. Secondly, investigating the mechanism by which transient changes in galanin through abdominal subcutaneous injection can lead to beneficial impacts even in an elevated background requires further research. Thirdly, studying the dynamic changes of galanin and its receptors during NAFLD/NASH progression may improve our understanding of our findings. Lastly, while NAFLD-related liver fibrosis models, such as long-term HFHCD or MCD, may be useful, NASH models have a major drawback, as they are unable to fully progress to severe steatohepatitis and advanced fibrosis, even after long-term feeding (11).

In conclusion, we uncovered a novel function of galanin in regulating the metabolic and pro-inflammatory phenotype of macrophage, which contributes to inhibiting NASH. This study showed that galanin caused a significant improvement in NASH features, including liver inflammation and fibrosis. All of these findings provide the enticing prospect of galanin as a promise for future practical applications in the control of NASH/NAFLD.

## Data availability statement

The original contributions presented in the study are included in the article/**Supplementary Material**. Further inquiries can be directed to the corresponding authors.

## Ethics statement

The studies involving human participants were reviewed and approved by Ethics Committee of Xinhua Hospital Affiliated to Shanghai Jiaotong University School of Medicine. The patients/participants provided their written informed consent to participate in this study. The Ethics approval number is XHEC-NSFC-2019-262. The animal study was reviewed and approved by Xinhua Hospital Ethics Committee Affiliated to Shanghai Jiaotong University School of Medicine. The Ethics approval number is XHEC-SHHDC-2020-063.

## Author contributions

YC and ZL designed and analyzed experimental data. LH, CH, and HW performed most of the experiments. NY and JZ helped in some animal experiments. LX and TG helped with some technical guidance. LH, TG, ZL, and YC prepared figures and wrote the manuscript. All authors contributed to the article and approved the submitted version.
