# Supplementary figures and images for: Galanin ameliorates liver inflammation and fibrosis in mice by activating AMPK/ACC signaling and modifying macrophage inflammatory phenotype
Source: Front Immunol. 2023 Apr 26;14:1161676. doi: 10.3389/fimmu.2023.1161676 (PMC10169601; doi:10.3389/fimmu.2023.1161676)

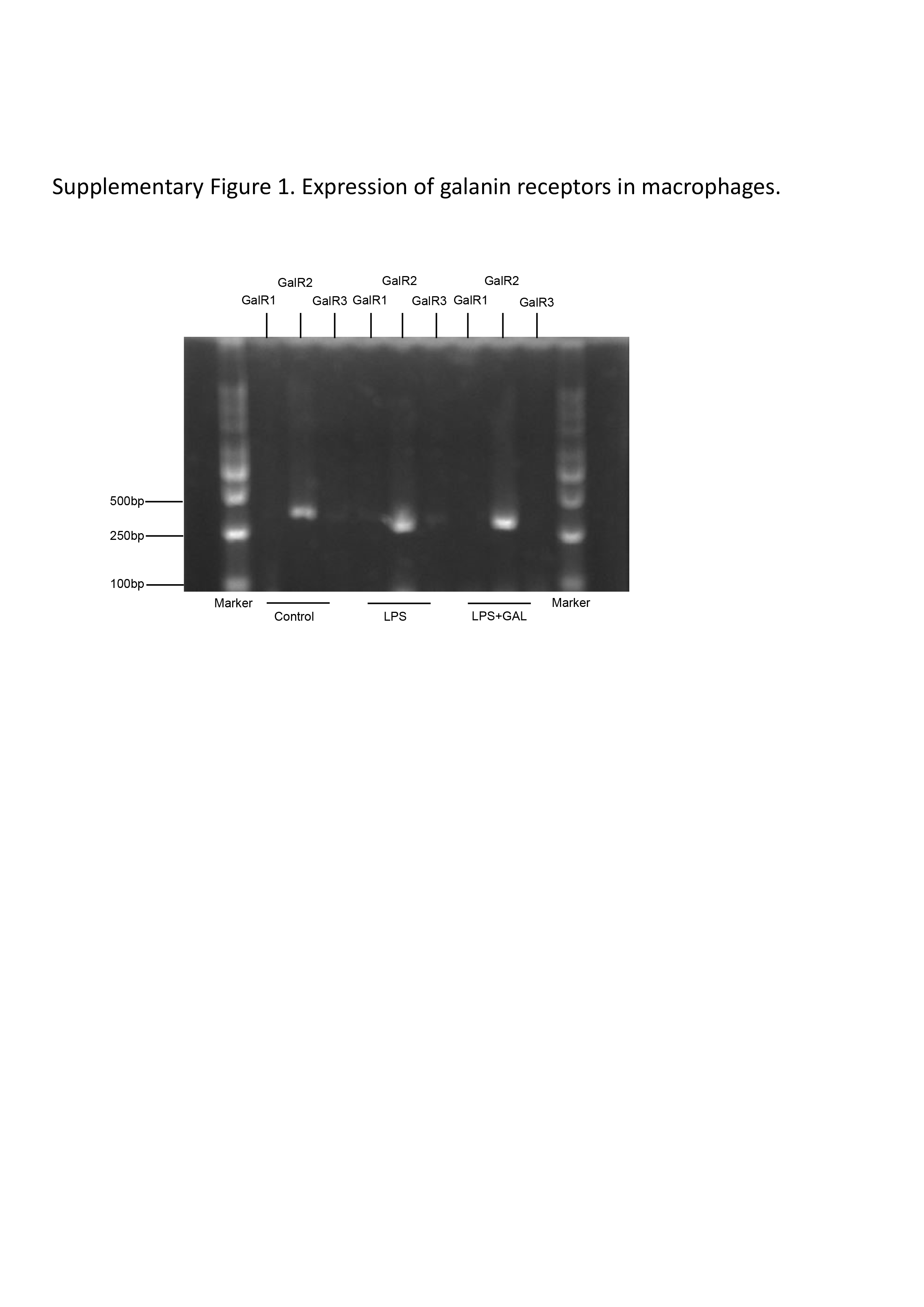

Supplement: Supplementary file 1 [file Image_1.tif]
