# Supplementary material for: Galanin ameliorates liver inflammation and fibrosis in mice by activating AMPK/ACC signaling and modifying macrophage inflammatory phenotype
Source: Front Immunol. 2023 Apr 26;14:1161676. doi: 10.3389/fimmu.2023.1161676 (PMC10169601; doi:10.3389/fimmu.2023.1161676)
Supplement: Supplementary file 2 [file Table_1.docx]

Supplementary Table S1. Primer for qRT-PCR

| **Number** | **Primer for qRT-PCR**  **(mouse)** | **Forward primer** | **Reverse primer** |
| --- | --- | --- | --- |
| 1 | TNF-α | TACCCCTGCCTGAGAGCAAT | CCACTTGGTGGTTTGTGAGTG |
| 2 | IL-1β | GGGCCTCAAAGGAAAGAATC | TACCAGTTGGGGAACTCTGC |
| 3 | IL-6 | TCTGCAAGAGACTTCCATCCA | AGTCTCCTCTCCGGACTTGT |
| 4 | GAPDH | TGCACCACCAACTGCTTAG | GGATGCAGGGATGATGTTC |
| 5 | CD68 | ACCTACATCAGAGCCCGAGT | GCATTTCCACAGCAGAAGC |
| 6 | MCP-1 | CTCTCTTCCTCCACCACCAT | GCTCTCCAGCCTACTCATTGG |
| 7 | CCR5 | GAGGAGCAGGGAGAACGAGT | GCAGTCAGGCACATCCATAG |
| 8 | α-SMA | AATGGCTCTGGGCTCTGTAA | TCTCTTGCTCTGGGCTTCAT |
| 9 | TGF-β1 | ATTCCTGGCGTTACCTTGG | AGCCCTGTATTCCGTCTCCT |
| 10 | COL-I | TGACTGGAAGAGCGGAGAGT | GACGGCTGAGTAGGGAACAC |
| 11 | COL-III | ATGGGTTTCCCTGGTCCTAA | TGCCTTGTAATCCTTGTGGA |
